# Invaders on the Wing

**DOI:** 10.1371/journal.pbio.1000398

**Published:** 2010-07-06

**Authors:** Mick N. Clout

**Affiliations:** School of Biological Sciences, University of Auckland, Auckland, New Zealand

**Figure pbio-1000398-g001:**
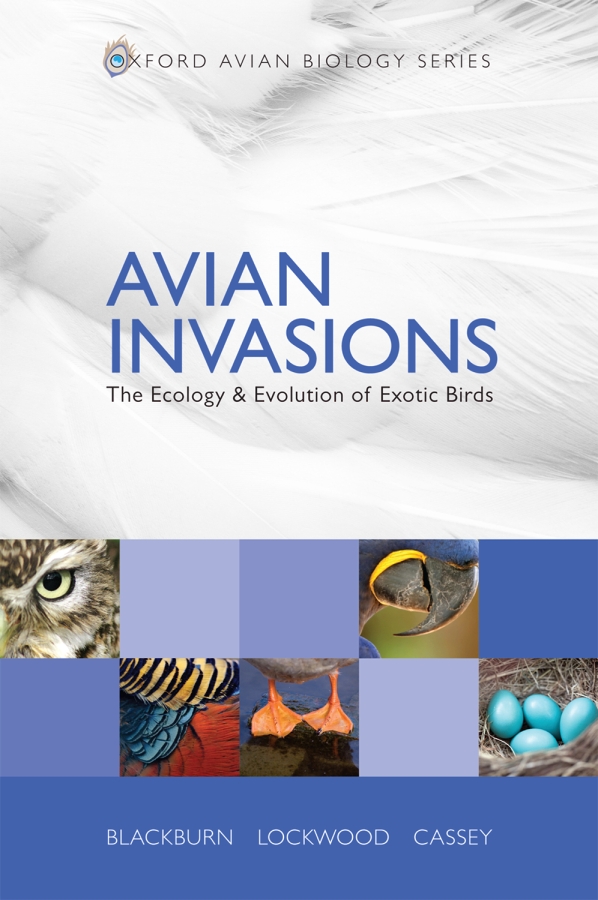
Blackburn TM, Lockwood JL, Cassey P (2009) Avian Invasions: The Ecology and Evolution of Exotic Birds. New York: Oxford University Press. 320p. ISBN (paperback): 978-0-19-923255-0. US$55.00.

[Fig pbio-1000398-g001]The deliberate or accidental introduction of species beyond their natural range has been one of the major consequences of the growth of trade, travel, and tourism in recent times. Many introduced species fail to establish or to spread significantly, but many others become successful invaders. Invasive alien species can transform natural ecosystems and cause extinctions of native species. As a result, invasive species are now recognized in the IUCN Red List as one of the main threats to native biodiversity worldwide. In some parts of the world, especially on oceanic islands and previously isolated landmasses, they are arguably now a more important threat than factors such as habitat loss or overexploitation, which tend to preoccupy conservationists in continental landscapes that humans have modified over thousands of years. For example, in New Zealand there is now a major “biosecurity” focus on preventing new invasions, eradicating introduced mammals from offshore islands, and managing a suite of invasive species on the mainland.

To deal effectively with invasive species, it is clearly important to understand the processes of biological invasion. If, by studying the “unnatural experiments” of past species introductions, insights can also gained into fundamental ecological processes, so much the better.

Although birds are very well-studied in many other contexts and are a common cause of conservation concern, they have received relatively little attention as biological invaders, compared with mammals, for example. This is despite the fact that the history and success or failure of avian introductions around the world is relatively well-documented. Since there is also a large amount of information on the distribution, ecology, and evolution of birds in general, this means that exotic birds provide excellent opportunities for examining the major stages of biological invasion and addressing important questions about the factors that facilitate invasions. The new book *Avian Invasions: The Ecology and Evolution of Exotic Birds*, by Tim Blackburn, Julie Lockwood, and Phillip Cassey, is therefore very timely and topical in its synthesis of the hitherto neglected subjects of the ecology and evolution of exotic birds.

The book is structured around widely accepted stages of the invasion pathway: transport and introduction, establishment, spread, and invasive impact. It concludes with chapters on the ecology, genetics, and evolution of exotic birds, and lessons learned from the study of avian invasions. Throughout the book, the authors not only critically review the existing literature on biological invasions in general and avian invasions in particular; they also make several excellent suggestions for future research.

The book starts with a brief but interesting history of bird introductions and a summary of the general invasion pathway. From the outset, the authors point out some of the problems in trying to draw scientific conclusions from historical data, which are often biased in terms of taxon, location, and time period. The bird species chosen for introduction reflected societal demands at the time, with the result that more than half of all known avian introductions come from only five families, which contain less than 15% of extant birds. Gamebirds, parrots, and passerines are over-represented among introduced birds because they were disproportionately selected for introduction as hunting quarry, as ornamental species, or as songbirds. Common bird species may also have been more likely to be successfully introduced; for example, common British birds were introduced to New Zealand in larger numbers. Overall, more than two thirds of bird introductions have been to islands, which comprise only a small fraction of the world's land area, and a disproportionate number of introductions have been made to Australia, New Zealand, and the Pacific Islands. Despite such shortcomings in the historical data, the authors illustrate throughout the book how the rigorous analysis of past introductions can still yield some powerful insights into invasion processes.

The first two stages of the invasion pathway are transport and introduction. These are considered together as the subject of the second chapter, with a focus on evidence for non-randomness in both stages. The authors point out that a consistent thread running through analyses of character selectivity is that the “availability” of a species is important for its success; large geographic range size and/or population size are predictors of transport and introduction probability.

The third stage of invasion is the crucial one of establishment, and this is the subject of chapters 3–5, which progressively consider the roles of contingency, species traits, and location in relation to establishment success. It is widely agreed that “propagule pressure” (usually defined as the number of individuals introduced) is an important determinant of success. The authors argue why this would be expected, relating it to the challenges faced by small populations in general and presenting evidence for the relationship between propagule pressure and establishment success. They then go on to consider the role of species-level traits, where previous studies have generally failed to find associations with establishment success. By framing this issue in the context of small population biology, they successfully argue that general conclusions can be drawn about the importance of factors such as population growth rates, Allee effects (reduced population growth at low densities), and abilities to cope with novelty, in relation to the likelihood of successful establishment. Chapter 5 discusses the importance of recipient location, including the historically controversial topics of invasibility and the role of competition in determining establishment success. The authors successfully revisit these arguments and also address the other important biotic interactions of predation, parasitism, and mutualisms (such as pollination and seed dispersal).

After establishment, the next phase of the invasion process is spread (which is discussed in Chapter 6). The authors review the general literature on geographic range expansion of invasive species and note the strong bias in substantial data from a few widespread and highly successful bird species (such as starlings and sparrows). Despite the taxonomic bias, this is a key area in which data on exotic birds have contributed substantially to our current understanding of one of the major stages of biological invasion.

In the second part of the book, the authors move beyond the invasion pathway to consider the ecology, genetics, and evolution of exotic birds, lessons learned from the study of avian invasions, and some of the exciting research opportunities that they present. They review information on interactions between exotic birds and native species and consider how comparing data on phenomena, such as species-area relationships and latitudinal gradients of exotic and native birds, can contribute to general ecological understanding. For example, whereas native bird species richness on islands is a negative function of distance to the mainland, exotic bird species richness is a positive function of this distance because of where exotic species were introduced. The authors then summarise what is known about the impacts of exotic birds on native species and communities (i.e., effectively the final stage of the invasion pathway). These impacts include competition with native birds, predation, mutualisms (such as pollination and seed dispersal), and the role of exotic birds in disease transmission. Classic examples include the pollination and dispersal of native and introduced plant species by exotic birds in many ecosystems and the devastating transmission of avian malaria and avian pox from exotic birds to vulnerable native bird species in Hawaii.

Chapters 8 and 9 take the longer-term, evolutionary perspective on the effects of successful avian invasions. Exotic birds have sometimes lost significant amounts of genetic variation during the introduction process, but in many cases there is no evidence of any such loss. There is, however, some evidence of evolution in phenotypes of exotic birds since they have established at new sites, despite the relatively short time frames involved. As is stressed by the authors, the “experiments in nature” represented by avian invasions, provide multiple (but so far under-utilized) opportunities for addressing basic questions in evolutionary ecology.

The authors conclude their book by stating that a coherent picture is beginning to emerge of how introduced bird species move through stages of the invasion process. Significant and consistent themes include the role of humans; interactions between event, species, and location; and the importance of both the stochastic and the deterministic in the establishment and spread of exotic birds. They strongly advocate the use of transport/introduction patterns as null hypotheses in invasion ecology, suggesting that ecological explanations are needed when there is deviation from these human-mediated patterns.

Overall, this is a comprehensive, up-to-date, and exciting book. It provides a much-needed stimulus for a greater focus on avian invasions and their effects on native biodiversity, but it also illustrates how the study of exotic birds can help to advance our general understanding of invasion ecology. I predict that it will become a classic text in invasion ecology, and I strongly commend it to all who are interested in this important and growing field.

